# Enhanced Compressive Strength of PVA/SA Composite Hydrogel by Highly Dispersed Hydroxyapatite Nanofibers

**DOI:** 10.3390/molecules30071631

**Published:** 2025-04-06

**Authors:** Shuochao You, Shan Zhang, Yahao Geng, Tianhao Wu, Guiyong Xiao

**Affiliations:** 1 Key Laboratory for Liquid-Solid Structural Evolution and Processing of Materials, Ministry of Education, Shandong University, Jinan 250061, China; cglfyscxz@163.com (S.Y.); shanzhang_sdu@163.com (S.Z.); 202234178@mail.sdu.edu.cn (Y.G.); 2928917256@gmail.com (T.W.); 2 School of Materials Science and Engineering, Shandong University, Jinan 250061, China

**Keywords:** hydroxyapatite nanofiber, hydrogel, mechanical property, hydrophilicity

## Abstract

Rapid functional soft tissue restoration has shown considerable promise as a framework for stability and coordination in the human body. Inspired by the anisotropic arrangement of structures with soft and hard phases in biological tissues, such as tendon, cartilage, and ligament, many methods have been used to fabricate composite hydrogels with appropriate mechanical properties. The development of a high-strength hydrogel with strong bioactivity remains a key barrier to replace soft tissues with comparable synthetic structures. In this study, a highly dispersed hydroxyapatite nanofiber (HANF) reinforced polyvinyl alcohol-sodium alginate (PVA/SA) composite hydrogel is prepared for soft tissue replacement. The effect of the addition of HANF on the microstructure and properties of composite hydrogel is also investigated. The results show that the PVA/SA hydrogel, after the incorporation of HANF, combines well with the PVA/SA hydrogel (HANF@PVA/SA). SEM morphologies show that dispersed HANF can enter the holes of the three-dimensional structure of the composite hydrogel. Additionally, the addition of HANF can enhance the compressive strength of the PVA/SA composite hydrogel from 4.66 MPa to 7.72 MPa. At the same time, the HANF@PVA/SA hydrogel maintains the same excellent hydrophilicity as the original PVA/SA hydrogel. Finally, cytotoxicity and live/dead cell staining tests also confirmed its excellent biocompatibility, demonstrating its tremendous potential for use in soft tissue repair.

## 1. Introduction

Soft tissue injuries are very common and frequently co-exist with other kinds of injuries. For instance, a significant number of related injuries (58.8%) are linked to pediatric proximal tibia fractures (PPTFs), especially tibial eminence fractures (TEFs 63.5%) and tibial plateau fractures (TPFs 100%) [1]. As is well known, autologous transplantation is the best way to repair soft tissues in the clinic. However, this procedure requires the removal of soft tissue from other areas of the body, which can result in additional harm to the patient. Xenogeneic tissue transplantation is intended to decellularize animal tissues, such as skin dermis, fascia, etc., while retaining the major components of the extracellular matrix (ECM). However, there are complications [2], such as pathogen transmission, and therefore, increased infection rates and immune rejection [3]. Furthermore, the main issues of soft tissue transplantation are donor difficulties and limited tissue sources [4,5]. Therefore, researchers are looking for suitable materials to replace injured soft tissues in clinical practice [6,7].

Hydrogel is a highly prominent material with a three-dimensional network structure made up of hydrated polymer chains. With a water content of up to 90% or more by weight, the features and topologies of hydrogel are comparable to those of ECMs [8], making it an ideal substrate for cell development and tissue regeneration. It can form a biocompatible hydration layer to provide the required humid environment for cell growth, while playing a supporting, protecting, and repairing role [9]. Because of its unique chemical and mechanical characteristics [10], hydrogel can accurately replicate the soft tissue found in the human body [11,12], such as ligament, muscle, and tendon. Hydrogel and its composites can improve cell adhesion, offer superior biocompatibility, and efficiently guarantee the flow of oxygen and nutrients [13]. Numerous reports on hydrogel-based soft tissue [14,15,16] restoration are now available. For instance, by encouraging the proliferation of skin cells and the deposition of collagen and other ECM proteins, a sodium alginate (SA) hydrogel scaffold has been utilized to regenerate skin tissue [17]. As a widely used hydrogel, SA has become an important candidate material [18,19] for soft tissue repair. It has the advantages of low cost, non-toxicity, easy processing, and rapid gelation, and its characteristics are similar to those of natural extracellular matrix, which can encapsulate cells in a highly hydrated three-dimensional environment. However, SA has poor mechanical properties and cannot provide sufficient mechanical support in a dynamic environment [20], which limits its clinical application. Physical blending modification [21] is one of the simplest, most economical, and most practical methods for creating novel polymeric composite materials. In principle, SA has a molecular structure of hydroxyl and carboxyl groups that easily form intermolecular hydrogen bonds, which greatly improves the mechanical properties of composites when combined with other polymers. The interaction between these functional groups contributes to improving the strength, flexibility, and surface performance of composite materials [22]. The hydroxyl groups in PVA and the carboxyl groups in SA generate ionic crosslinks via hydrogen bonding, resulting in an ionically crosslinked network. This crosslinked three-dimensional structure has great hydration capacity, and the hydrogen bonds increase the stability and mechanical strength of hydrogel. Therefore, PVA is an ideal material for mixing with SA [23]. However, in some complex application scenarios, for instance, in parts of the body which undergo regular movement, complicated stresses might induce hydrogel injury [24]; as such, the mechanical properties of PVA-SA hydrogels still have shortcomings.

The introduction of nanofibers into hydrogels [25] provides a feasible way to solve the insufficient mechanical properties of hydrogels. Hydroxyapatite (HA) is the principal inorganic component of vertebrate bones and teeth and has broad application prospects in the field of biomedicine because of its excellent biological activity [26,27]. Due to its high aspect ratio, HANF possesses excellent mechanical properties, and controlling its aspect ratio could provide new possibilities for designing materials with unique mechanical properties. It has also been reported that hydroxyapatite fibers (HANF) play a great role in enhancing the mechanical properties of SA hydrogels [28]. 

**Figure 1:**
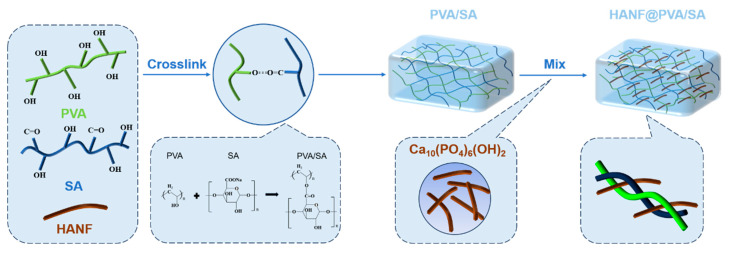
Schematic illustration of the synthetic mechanism and structure of PVA-SA/HANF hy-drogel.

In this work, highly dispersed HANF was added to PVA/SA blends, and HANF@PVA/SA composite hydrogels were prepared by a repeated freeze–thaw method. In order to investigate the effect of the HANF content on the morphology, compressive strength, and surface properties of HANF@PVA/SA composite hydrogels, three groups of samples with different contents of HANF were prepared. The results show that the addition of highly dispersed HANF to PVA/SA hydrogels can change the three-dimensional porous structure and significantly improve the compressive properties, although their wettability showed no significant changes. Due to its excellent mechanical properties and biocompatibility, the fabricated HANF@PVA/SA hydrogel has broad application prospects in biomedical fields such as soft tissue repair. Figure 1 illustrates the schematic of HANF embedded in the hydrogel network.

## 2. Results and Discussion

### 2.1. Functional Group Analysis of HANF@PVA/SA Hydrogel

**Figure 2:**
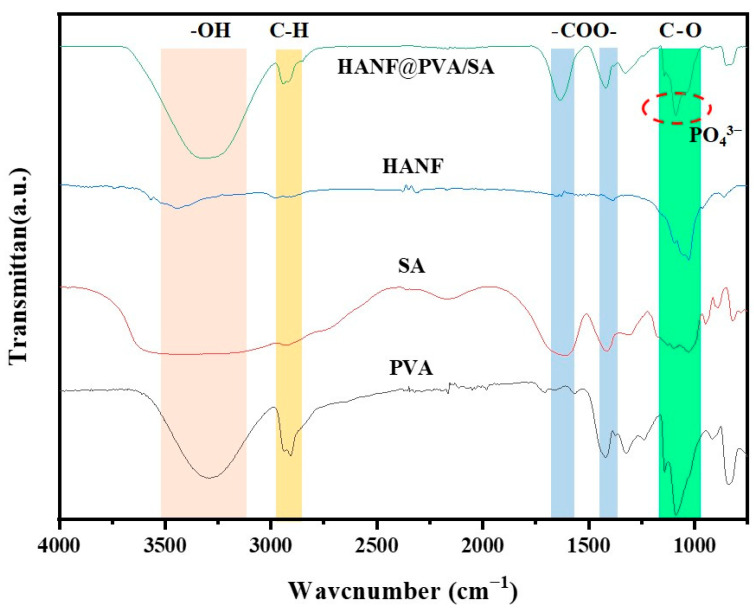
FTIR spectra of PVA, SA, HANF, and HANF@PVA/SA hydrogels.

Figure 2 shows the FTIR spectra of the HANF@PVA/SA composite hydrogel and its constituents. The results show that HANF can fully integrate into the PVA/SA matrix. In the HANF@PVA/HA hydrogels, the absorption peaks of -OH were concentrated in the area below 3331 cm^−1^, and the absorption peak of C-H appeared at 2934 cm^−1^. Additionally, 1636 cm^−1^ and 1418 cm^−1^ correspond to the asymmetric vibrations and symmetric vibrations of -COO-, while 1327 cm^−1^ is the C-O bond of alcohol hydroxyl groups [29,30]. As shown in Figure 2, the FTIR spectra of HANF showed that the absorption peaks of PO_4_^3−^ were also near 1088 cm^−1^, the final infrared spectrum of HANF@PVA/SA that we obtained. We speculate that in the process of hydrogel synthesis, PVA first physically cross-linked with SA, and PVA supplied a significant number of hydroxyl and hydrogen bonds with SA polymer chains to construct a three-dimensional network structure with a certain mechanical strength. HANFs were disseminated throughout the hydrogel network, where they could act as the physical crosslinking point to increase the strength of the composite hydrogel and function as a fiber-reinforced hydrogel.

### 2.2. Morphology of HANF@PVA/SA Hydrogel

**Figure 3:**
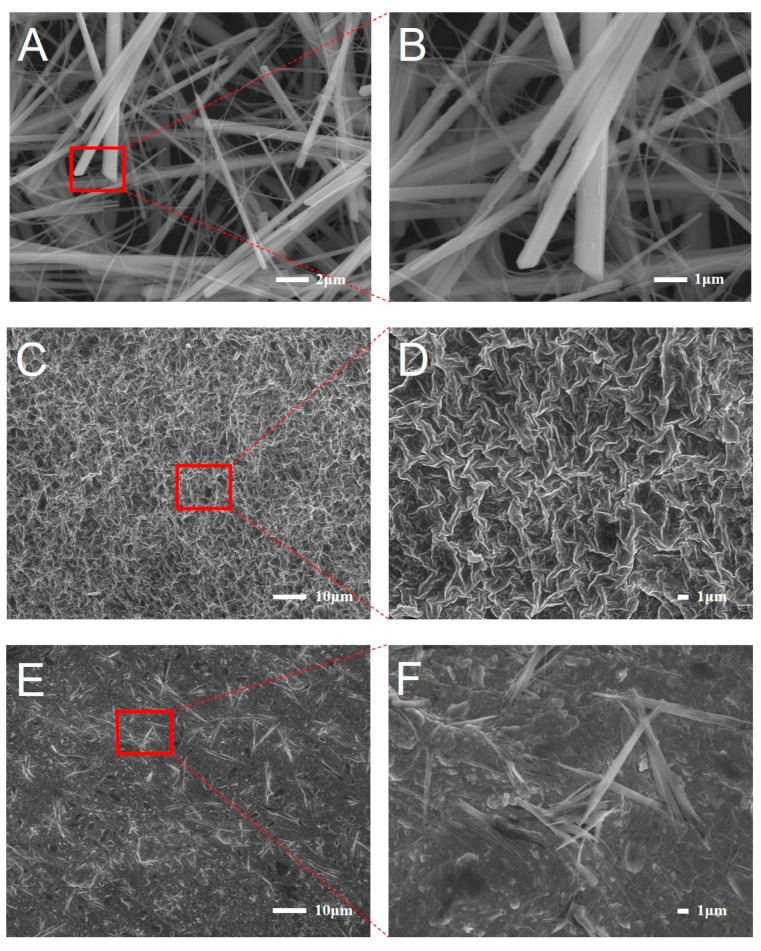
SEM micrographs of the dispersed HANFs (**A**,**B**), PVA/SA composite hydrogel (**C**,**D**), and HANF@PVA/SA hydrogel (**E**,**F**). (**B**,**D**,**F**) are high magnifications of (**A**,**C**,**E**), respectively.

The addition of highly dispersed HANF into the PVA/SA hydrogel significantly affected the morphology of the composite. Figure 3 depicts the morphologies of the PVA/SA hydrogel and HANF@PVA/SA hydrogel. SEM micrographs of HANF prepared by the cal-cium oleate precursor solvothermal method are given in Figure 3A,B. The results clearly indicate that the hydroxyapatite fibers appeared as short forms, with the length of the HANF added to the hydrogel ranging between 10–15 µm. In addition, the as-prepared HANFS displayed high dispersion, which can be explained by the small diameters and short lengths. Figure 3C,D show the surface morphology of the as-prepared PVA/SA composite hydrogel. The SEM data indicated that the PVA/SA composite could form a 3D network with a uniform hole structure, which is favorable for the cell growth. We assume that the creation of holes in the hydrogel was caused by the fact that both PVA and SA are hydrophilic. During the crosslinking of PVA and SA, hydrogen bonds were generated to produce a three-dimensional network structure, which was then embedded with a large number of water molecules. The freeze–thaw process produced a bloated three-dimensional structure. Compared with the PVA/SA hydrogel, the three-dimensional porous structure of HANF@PVA/SA disappeared, as shown in Figure 3E,F. When dispersed HANFs were incorporated into PVA/SA, they integrated effectively with the composite hydrogel, displacing a portion of the water originally present in the structure and occupying its spatial position. Furthermore, the dispersed HANFs and dense structure of the HANF@PVA/SA hydrogel also enhanced its compressive strength by distributing the load and preventing crack propagation [31]. High magnification images prove that the HANFs were evenly interlaced in the PVA/SA hydrogel matrix, which further confirms our conjecture on the mechanism of fiber-reinforcement.

### 2.3. Compressive Performance of HANF@PVA/SA Hydrogel

**Figure 4:**
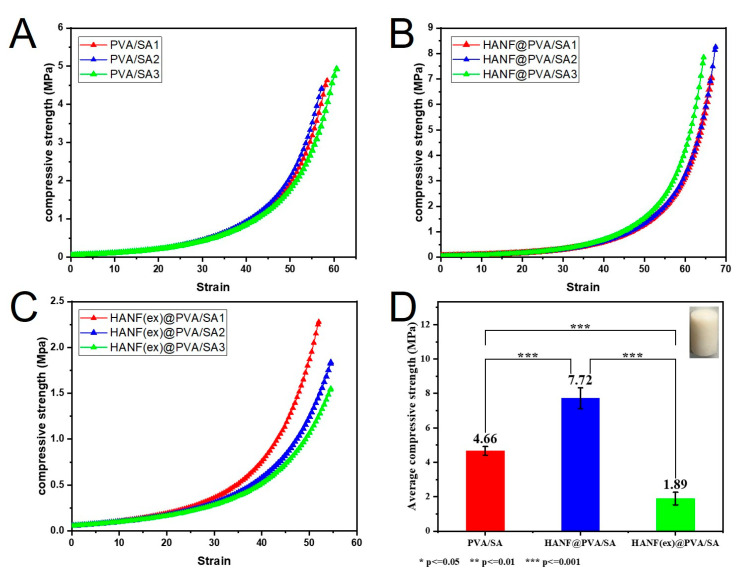
Compressive strength of the freeze dried HANF@PVA/SA hydrogel with different mass ratios of HANF: (**A**) HANF; (**B**) A small amount of HANF; (**C**) Excessive HANF (**D**) The average compressive strength of HANF@PVA/SA hydrogels with different HANF mass ratios.

To investigate the influence of HANF on the compressive properties of PVA/SA hy-drogels, three types of samples are prepared: composite PVA/SA hydrogels without HANF, HANF@PVA/SA hydrogels with low HANF content, and HANF(ex)@PVA/SA hydrogels with high HANF content. Figure 4 illustrates the compressive strength of the three samples. Compared with that of PVA/SA sample, the compressive property of the HANF@PVA/SA hydrogel was significantly better. The average compressive strength increased significantly from 4.66 MPa to 7.72 MPa, with highly significant differences observed among the three materials (*p* ≤ 0.001), demonstrating that HANFs effectively enhanced the strength of PVA/SA hydrogels. This result is consistent with our previous hypothesis, i.e., that HANFs would enter the three-dimensional holes of the PVA/SA hydrogel. The hydrogel with an excess of HANFs (HANF(ex)@PVA/SA), on the other hand, revealed poor compressive strength in the compression tests. It is speculated that the excess HANFs may have agglomerated, thus splitting the matrix and resulting in a decrease in compression performance.

### 2.4. Wettability of HANF@PVA/SA Hydrogel

**Figure 5:**
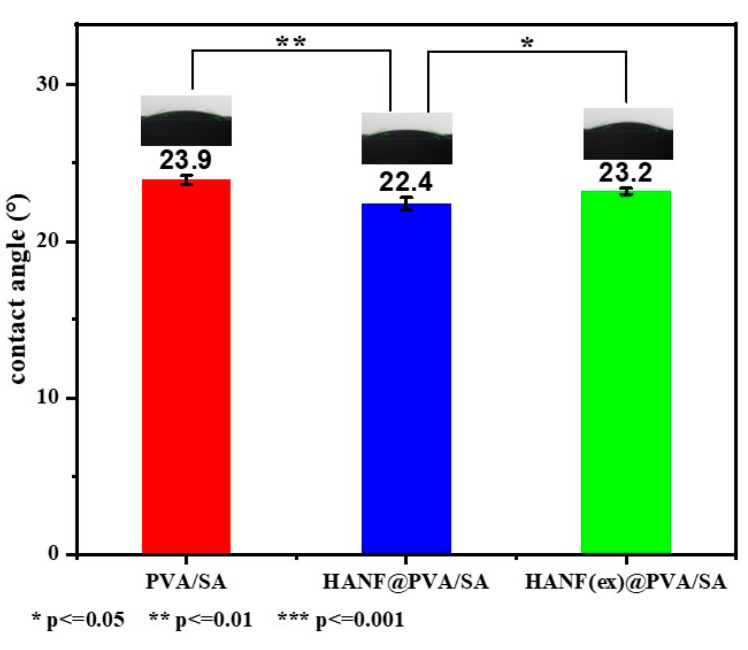
Wetting angle of HANF@PVA/SA hydrogel.

In the field of soft tissue repair, the wettability of hydrogels is one of the most important factors. For example, as a skin wound repair material, maintaining a suitably moist environment is conducive to the enhancement of immune cell function and the mi-gration speed of epidermal cells, thereby accelerating wound healing. Wetting angle pictures of the three groups of samples are shown in Figure 5. The results show that all the samples had excellent hydrophilicity and exhibited no obvious changes in wetting angle before or after adding HANFs to the PVA/SA hydrogel; this was likely because wettability is mainly affected by the physical structure and chemical composition of the hydrogel surface. The addition of HANFs did not change the three-dimensional network structure or the chemical composition.

### 2.5. Biocompatibility of HANF@PVA/SA Hydrogel

**Figure 6:**
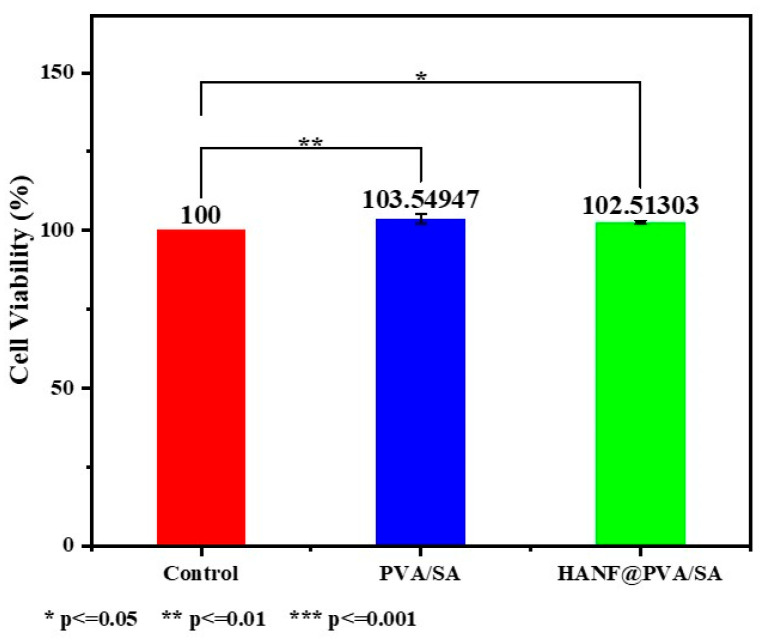
CCK-8 cytotoxicity of HANF@PVA/SA hydrogel.

In this study, the biocompatibility of PVA/SA and HANF@PVA/SA materials was evaluated using CCK-8 cytotoxicity assays and live/dead staining with confocal microscopy (Figure 6). The CCK-8 results revealed that the cell viability in the PVA/SA group and the HANF@PVA/SA group was 103.55% and 102.51%, respectively, both slightly higher than that of the control group (100%). These findings indicate that neither material exhibited significant cytotoxicity and may even promote cell proliferation to some extent. Moreover, the minimal difference in cell viability between the PVA/SA and HANF@PVA/SA groups (103.55% vs. 102.51%) suggests that the incorporation of HANF did not significantly alter the biological activity of the PVA/SA matrix.

**Figure 7:**
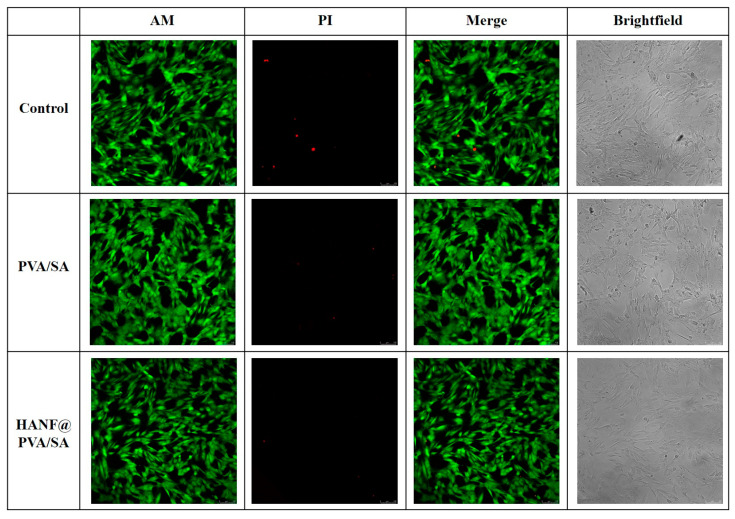
Confocal microscopy images of live/dead cell staining.

To validate the reliability of the CCK-8 results, live/dead staining was performed to assess cell viability. Confocal microscopy images(Figure 7) demonstrated that the number of dead cells in the experimental groups was negligible, with cells exhibiting intact morphology and uniform distribution. The proportion of live cells in both experimental groups exceeded 95%, consistent with the high cell viability observed in the CCK-8 assay. Qualitative analysis of the live/dead staining further confirmed the excellent biocompatibility of both the PVA/SA and HANF@PVA/SA materials.

For a more in-depth analysis, statistical comparisons were conducted to evaluate the differences between groups. The results showed no statistically significant difference (*p* > 0.05) between the PVA/SA and HANF@PVA/SA groups, providing further evidence that the addition of HANF did not significantly affect the biocompatibility of the PVA/SA matrix. Additionally, the live/dead staining images provided qualitative evidence of healthy cell morphology, with minimal apoptotic or necrotic cells, aligning well with the quantitative CCK-8 data.

These results demonstrate that both PVA/SA and HANF@PVA/SA materials exhibit favorable biocompatibility, showing great potential for applications in soft tissue repair.

## 3. Materials and Methods

### 3.1. Materials

The oleic acid used in the experiment was purchased from Shanghai Aladdin Bio-chemical Technology Co., Ltd. (Shanghai, China). Anhydrous ethanol (AR), sodium hydroxide (NaOH, AR), calcium chloride (CaCl_2_, AR), sodium dihydrogen phosphate dihydrate (NaH_2_PO_4_·2H_2_O, AR), polyvinyl alcohol (PVA), and sodium alginate (SA) were purchased from Sinopharm Chemical Reagent Co., Ltd. (Shanghai, China). All chemical reagents were used without any further purification.

### 3.2. Preparation of Highly Dispersed HANF

The products of HANF were prepared by the solvothermal method. First, we combined oleic acid (24 g), anhydrous ethanol (22 g), and deionized water (20 mL) in a sealed container at room temperature and stirred constantly until a suspension formed. A mixed solution was then created by gradually adding sodium hydroxide solution (40 mL, 1.28 M), calcium chloride solution (40 mL, 0.2 M), and disodium hydrogen phosphate solution (40 mL, 0.2 M) to the suspension at a rate of 70 mL/h. Then, using an air-drying oven, the mixed solution was heated in a 200 mL hydrothermal reaction tank at 180 °C for 30 h and subsequently cooled to room temperature. Finally, the product was washed three times with anhydrous ethanol and deionized water and then centrifuged to obtain HANF.

### 3.3. Preparation of HANF@PVA/SA Hydrogel

PVA (10 g) was added to 88 mL of deionized water, vigorously stirred at 85 °C for 3 h, and naturally cooled to 50 °C. We subsequently added SA (2 g) and continued stirring for 1 h. The composite PVA/SA hydrogel was produced after deposition for 12 h. Simul-taneously, the scalar quantitative HANF was placed into a centrifuge tube containing 15 mL of anhydrous ethanol (AR) and ultrasonically dispersed for 45 min. Next, the HANF was centrifuged for 3 min. Then, the dispersed HANF was slowly mixed into the composite PVA/SA hydrogel, frozen at low temperature for 8 h, and dissolved for 4 h. The above steps were repeated three times. Finally, the HANF@PVA/SA was cross-linked using 3% CaCl_2_ solution for 24 h. Cylinder compressed samples with 1.5 cm diameter and 3 cm height were prepared for mechanical performance tests. In that compressive test, 0.1 g and 0.4 g HANF were added to 100 mL PVA/SA hydrogels, named HANF@PVA/SA and HANF(ex)@PVA/SA, respectively.

### 3.4. Materials Characterization

Fourier transform infrared spectroscopy (FTIR) (Nexus 670, Thermo Nicolet, Wal-tham, MA, USA) was utilized to identify the functional groups in the products in the 4000–500 cm^−1^ range. The morphologies of the samples were examined using a field emission scanning electron microscope (FESEM) (SU70, Hitachi, Japan). All test samples were discs with a diameter of 1.5 cm and a thickness of 1 mm. Prior to testing, the samples were subjected to freeze-drying at 0 °C for 48 h. The compression performance was tested using a microcomputer-controlled electronic universal testing machine (ZCW-W10KN, Jinan Testing Instrument Co., Ltd., Jinan, China). The compression test samples were cylindrical with a diameter of 1.5 cm and a height of 3 cm. For each hydrogel group, five replicate samples were prepared. The contact angle was measured with a contact angle goniometer (DSA100S, Krüss, Hamburg, Germany). For the contact angle test, three replicate samples were prepared for each group.

### 3.5. Materials Biocompatibility

CCK-8 (Beyotime, C0037) was employed to assess the cytotoxicity of the materials. Specifically, mouse embryonic mesenchymal stem cells were seeded in 96-well plates at a density of 4 × 10^3^ cells per well and cultured for 24 h. The materials were then added to the wells, and after 48 h of incubation, 10 µL of CCK-8 solution was added to each well. The plates were further incubated for 4 h at 37 °C, and add 100 μL of Formazan solution was added to each well and mixed gently. Then, we continued incubating in the cell culture incubator until the Formazan had completely dissolved, as observed under a standard optical microscope. The absorbance was measured at 450 nm using a microplate reader to determine cell viability. Three groups were tested in triplicate: a control group, the PVA/SA hydrogel extract, and the HANF@PVA/SA hydrogel extract.

The examination of live and dead cell staining was performed using Laser Scanning Confocal Microscopy (LSCM) (Leica, SP8 DIVE). The same cell culture method as CCK-8 was employed. After incubation, the cells were stained with a live/dead viability assay kit, i.e., Calcein (AM) for live cells and propidium iodide (PI) for dead cells. The stained cells were then imaged using LSCM to visualize and quantify the live and dead cell populations. Three groups were tested in triplicate: a control group, the PVA/SA hydrogel extract, and the HANF@PVA/SA hydrogel extract.

### 3.6. Statistical Analysis

All quantitative results were derived from a minimum of three independent samples. Data visualization was performed, and statistical analysis was conducted using Origin 2021. Unpaired *t*-tests were employed to compare differences between two groups, with statistical significance defined as * *p* ≤ 0.05, ** *p* ≤ 0.01, and *** *p* ≤ 0.001.

## 4. Conclusions

In this work, a highly dispersed HANF-reinforced PVA/SA composite hydrogel (HANF@PVA/SA) was successfully prepared. The addition of HANF can obviously influence the microstructure and properties of a composite hydrogel. The FTIR results prove that the dispersed HANFs can fully integrated into a PVA/SA composite hydrogel. The dispersed HANFs combined well with the composite hydrogel and filled the holes in the hydrogel insight structure, resulting in the formation of a relatively dense structure. Furthermore, the dispersed HANFs and dense structure of the HANF@PVA/SA hydrogel also enhanced its compressive strength from 4.66 MPa to 7.72 MPa. At the same time, the HANF@PVA/SA hydrogel maintained the same excellent hydrophilicity as the original PVA/SA hydrogel and showed no obvious change in wetting angle before or after adding the HANFs. The strong correlation between the CCK-8 cytotoxicity test and the live/dead staining outcomes provides robust evidence for the superior biocompatibility of the HANF@PVA/SA hydrogel. Given the hydrogel’s excellent compressive properties and good biocompatibility, we believe that the HANF@PVA/SA hydrogel, which exhibits high compressive strength, shows promising potential for applications in skin repair. The addition of high dispersed HANFs to PVA/SA hydrogel offers the possibility of a novel composite hydrogel material for soft tissue repair.

## Data Availability

The raw data supporting the conclusions of this article will be made available by the authors on request.

## References

[1] Sanders E., Policicchio A.L., Phillips L. (2023). High incidence of soft tissue injury in pediatric proximal tibia fractures: A systematic review. Arthrosc. Sports Med. Rehabil..

[2] Stosich M.S., Moioli E.K., Wu J.K., Lee C.H., Rohde C., Yoursef A.M., Ascherman J., Diraddo R., Marion N.W., Mao J.J. (2009). Bioengineering strategies to generate vascularized soft tissue grafts with sustained shape. Methods.

[3] Badylak S.F. (2002). The extracellular matrix as a scaffold for tissue reconstruction. Seminars in Cell & Developmental Biology.

[4] Gilbert T.W. (2012). Strategies for tissue and organ decellularization. J. Cell. Biochem..

[5] Xue Y., Riva N., Zhao L., Shieh J.S., Chin Y.T., Gatt A., Guo J.J. (2023). Recent advances of exosomes in soft tissue injuries in sports medicine: A critical review on biological and biomaterial applications. J. Control Release.

[6] Xu S., Ahmed S., Momin M., Hossain A., Zhou T. (2023). Unleashing the potential of 3D printing soft materials. Device.

[7] Van Nieuwenhove I., Tytgat L., Ryx M., Blondeel P., Stillaert F., Thienpont H., Ottevaere H., Dubruel P., Van Vlierberghe S. (2017). Soft tissue fillers for adipose tissue regeneration: From hydrogel development toward clinical applications. Acta Biomater..

[8] Lee K.Y., Mooney D.J. (2001). Hydrogels for tissue engineering. Chem. Rev..

[9] Barros D., Freitas Amaral I., Paula Pego A. (2015). Biomimetic synthetic self-assembled hydrogels for cell transplantation. Curr. Top. Med. Chem..

[10] Drury J.L., Mooney D.J. (2003). Hydrogels for tissue engineering: Scaffold design variables and applications. Biomaterials.

[11] Seliktar D. (2012). Designing cell-compatible hydrogels for biomedical applications. Science.

[12] Annabi N., Tamayol A., Uquillas J.A., Akbari M., Bertassoni L.E., Cha C., Camci-Unal G., Dokmeci M.R., Peppas N.A., Khademhosseini A. (2014). 25th anniversary article: Rational design and applications of hydrogels in regenerative medicine. Adv. Mater..

[13] Zhu J., Marchant R.E. (2011). Design properties of hydrogel tissue-engineering scaffolds. Expert Rev. Med. Devices.

[14] Chatterjee S., Upadhyay P., Mishra M., Srividya M., Akshara M., Kamali N., Zaidi Z.S., Iqbal S.F., Misra S.K. (2020). Advances in chemistry and composition of soft materials for drug releasing contact lenses. RSC Adv..

[15] Van Vlierberghe S., Dubruel P., Schacht E. (2011). Biopolymer-based hydrogels as scaffolds for tissue engineering applications: A review. Biomacromolecules.

[16] Pahlevanzadeh F., Mokhtari H., Bakhsheshi-Rad H.R., Emadi R., Kharaziha M., Valiani A., Poursamar S.A., Ismail A.F., Ramakrishna S., Berto F. (2020). Recent trends in three-dimensional bioinks based on alginate for biomedical applications. Materials.

[17] Balakrishnan B., Mohanty M., Umashankar P.R., Jayakrishnan A. (2005). Evaluation of an in situ forming hydrogel wound dressing based on oxidized alginate and gelatin. Biomaterials.

[18] Tarassoli S.P., Jessop Z.M., Jovic T., Hawkins K., Whitaker I.S. (2021). Candidate bioinks for extrusion 3D bioprinting—A systematic review of the literature. Front. Bioeng. Biotechnol..

[19] Schütz K., Placht A.M., Paul B., Brüggemeier S., Gelinsky M., Lode A. (2017). Three-dimensional plotting of a cell-laden alginate/methylcellulose blend: Towards biofabrication of tissue engineering constructs with clinically relevant dimensions. J. Tissue Eng. Regen. Med..

[20] Lee K.Y., Mooney D.J. (2012). Alginate: Properties and biomedical applications. Prog. Polym. Sci..

[21] Wei Q., Zhou J., An Y., Li M., Zhang J., Yang S. (2023). Modification, 3D printing process and application of sodium alginate based hydrogels in soft tissue engineering: A review. Int. J. Biol. Macromol..

[22] Mehrjou A., Hadaeghnia M., Namin P.E., Ghasemi I. (2024). Sodium alginate/polyvinyl alcohol semi-interpenetrating hydrogels reinforced with PEG-grafted-graphene oxide. Int. J. Biol. Macromol..

[23] Jiang X., Xiang N., Zhang H., Sun Y., Lin Z., Hou L. (2018). Preparation and characterization of poly (vinyl alcohol)/sodium alginate hydrogel with high toughness and electric conductivity. Carbohydr. Polym..

[24] Adelnia H., Ensandoost R., Moonshi S.S., Gavgani J.N., Vasafi E.I., Ta H.T. (2022). Freeze/thawed polyvinyl alcohol hydrogels: Present, past and future. Eur. Polym. J..

[25] Hao L., Liang S., Han Q., Jing Y., Li J., Li Q., Wang A., Bai S., Yin J. (2023). Ultralong hydroxyapatite nanowires-incorporated dipeptide hydrogel with enhanced mechanical strength and superior in vivo osteogenesis activity. Colloids Surf. A Physicochem. Eng. Asp..

[26] Chen F., Zhu Y.J. (2014). Multifunctional calcium phosphate nanostructured materials and biomedical applications. Curr. Nanosci..

[27] Lu B.Q., Zhu Y.J., Chen F. (2014). Highly flexible and nonflammable inorganic hydroxyapatite paper. Chemistry.

[28] Jiang Y.Y., Zhu Y.J., Li H., Zhang Y.G., Shen Y.Q., Sun T.W., Chen F. (2017). Preparation and enhanced mechanical properties of hybrid hydrogels comprising ultralong hydroxyapatite nanowires and sodium alginate. J. Colloid Interface Sci..

[29] Kwon O.H., Kim J.O., Cho D.W., Kumar R., Baek S.H., Kurade M.B., Jeon B.H. (2016). Adsorption of As (III), As (V) and Cu (II) on zirconium oxide immobilized alginate beads in aqueous phase. Chemosphere.

[30] Hu T., Liu Q., Gao T., Dong K., Wei G., Yao J. (2018). Facile preparation of tannic acid-poly (vinyl alcohol)/sodium alginate hydrogel beads for methylene blue removal from simulated solution. ACS Omega.

[31] Yue Y., Han J., Han G., French A.D., Qi Y., Wu Q. (2016). Cellulose nanofibers reinforced sodium alginate-polyvinyl alcohol hydrogels: Core-shell structure formation and property characterization. Carbohydr. Polym..

